# Mindfully missing myself: Induced mindfulness causes alienation among poor self-regulators

**DOI:** 10.1371/journal.pone.0303505

**Published:** 2024-05-21

**Authors:** Niyati Thakur, Nicola Baumann

**Affiliations:** Department of Differential Psychology, Personality Psychology and Diagnostics, University of Trier, Trier, Germany; Iowa State University, UNITED STATES

## Abstract

Mindfulness is a popular technique that helps people to get closer to their self. However, recent findings indicate that mindfulness may not benefit everybody. In the present research, we hypothesized that mindfulness promotes alienation from the self among individuals with low abilities to self-regulate affect (state-oriented individuals) but not among individuals with high abilities to self-regulate affect (action-oriented individuals). In two studies with participants who were mostly naïve to mindfulness practices (70% indicated no experience; *N*_1_ = 126, 42 men, 84 women, 0 diverse, aged 17–86 years, *M*_age_ = 31.87; *N*_2_ = 108, 30 men, 75 women, 3 diverse, aged 17–69 years, *M*_age_ = 28.00), we tested a mindfulness group (five-minute mindfulness exercise) against a control group (five-minute text reading). We operationalized alienation as lower consistency in repeated preference judgments and a lower tendency to adopt intrinsic over extrinsic goal recommendations. Results showed that, among state-oriented participants, mindfulness led to significantly lower consistency of preference judgments (Study 1) and lower adoption of intrinsic over extrinsic goals (Study 2) compared to text reading. The alienating effect was absent among action-oriented participants. Thus, mindfulness practice may alienate psychologically vulnerable people from their self and hamper access to preferences and intrinsic goals. We discuss our findings within Personality-Systems-Interactions (PSI) theory.

*“If you know yourself, then you’ll not be harmed by what is said about you”*

[1]

## 1. Introduction

Aristotle’s quote *“knowing yourself is the beginning of all wisdom”* highlights that ideas related to knowing oneself are quite pertinent to well-being. However, people’s conscious self-knowledge usually does not cover the full spectrum of experience and has to be distinguished from the ‘true’ self [2]. The self can be defined as a complex knowledge structure comprising personal needs, emotions, preferences, goals, and values which operates largely at an implicit level [3,4]. The self is a vital source of psychological functioning [5]. However, access to the self gets blocked, for example, if people are overwhelmed by external pressure, stress, and negative affect [6]. This makes the ability to self-regulate negative affect an important resource against alienation from the self. However, not everybody has equal resources.

The disposition of action versus state orientation explains individual differences in the ability to self-regulate affect [3,7,8]. A plethora of findings show that, when confronted with stressful conditions, action-oriented people are able to down-regulate negative affect and maintain volitional control over their thoughts and actions, whereas state-oriented people are unable to down-regulate negative affect and get stuck in uncontrollable rumination [9–11]. This unattenuated negative affect makes state-oriented people more vulnerable towards losing touch with their *self* [6,12]. For example, under stressful conditions, state-oriented people have been found to show lower consistency in preference judgments and confuse external assignments as self-selected goals (for an overview, see 9). Since the *self* represents personal needs, emotions, preferences, and goals [3,4], lower consistency in preference judgments and confusing external assignments as self-selected goals indicates that state-oriented individuals lost access to their *self*. Therefore, an important question is how self-access can be restored among state-oriented people.

Mindfulness seems like a promising remedy as mindfulness has shown positive effects especially in terms of better emotion regulation [13,14] and stress regulation [15,16]. However, the idea that mindfulness always acts as a remedy is a “false myth”. Recent findings show that mindfulness can even be harmful and promote alienation among state-oriented people [17]. In the upcoming paragraphs, we elaborate on state-oriented people’s vulnerability towards alienation, review findings on mindfulness, and explain our hypothesis that mindfulness might not help but even hurt state-oriented people.

### 1.1. State-oriented people are vulnerable towards alienation

The theoretical foundation of action-state orientation is elaborated in the theory of Personality-Systems-Interactions (PSI; 603,4,11). PSI theory defines central cognitive systems and elaborates in detail how the regulation of affect influences their flexible interplay that is needed for adaptive personality functioning. Furthermore, PSI theory elaborates how action- and state-oriented individuals differ in their dispositional ability to self-regulate affect. Action versus state orientation is an intuitive, self-confrontational, and efficient type of coping with negative affect that is distinct from reappraisal [12], self-esteem [18,19], and self-efficacy [20,21].

Many findings support the assumption that state- compared to action-oriented people are indeed less able to self-regulate affect [22, 2018; 8,10,12]. For example, state-oriented students were unable to down-regulate negative affect when exams came closer, whereas action-oriented students successfully did [23]. This self-regulatory deficit has been shown to influence a broad range of life domains like psychological well-being [24], sleep quality [25], relationship satisfaction [26], and educational achievement [27].

Importantly, the range of adverse effects extends to indicators of self-access. Previous findings show that, under negative affect, state-oriented people became inconsistent in judging their preferences (e.g., for abstract art and soft drinks; 28), confused assigned and self-selected goals [6,29], and chose goals that did not match their implicit motives [23,24]. Action-oriented individuals, in contrast, did not show these signs of alienation and maintained self-access [12,30,31]. It is important to note that state-oriented individuals’ judgment inconsistencies, goal confusions, and motive-goal incongruences are markers of a broader tendency towards alienation from the *self*. Although they may seem like trivial outcomes in themselves, they do have profound consequences for psychological well-being and health such as higher psychosomatic symptoms [24], unhealthy eating behavior [32], and lower identity status [33].

Therefore, state-oriented people’s vulnerability towards alienation makes it imperative to find tools that can help to restore self-access. One promising resort is mindfulness. A huge body of research shows that practicing mindfulness has many beneficial effects and positively influences one’s self-view and self-regulation [34,35]. However, other findings indicate that mindfulness does not help everybody and may even hurt some people [36–39].

### 1.2. Does mindfulness help or hurt?

Mindfulness is a specific quality of awareness where people pay attention to all body and mind experiences in an open, non-judgmental, and accepting manner [40–42]. Mindfulness is associated with diverse benefits for psychological functioning. For example, practicing mindfulness has been found to improve emotion regulation [43], attention [44], emotional and psychological well-being [34,45], stress, depressive symptoms, anxiety, and rumination [16,46]. Furthermore, mindfulness practices induce a brain state that improves the resolution of conflict [47], helps coping with pain [48], improves relationship quality [49], and enhances social quality of life [50]. These findings suggest that mindfulness practices may also benefit state-oriented people.

In contrast, several studies also point to a negative side of mindfulness. For example, mindfulness training and high levels of self-focused attention have been associated with psychopathology [51], negative affect [52], and increased psychophysiological stress responses [35]. Other findings show that mindfulness also has negative effects on the self as indicated by cognitive anomalies [53], dissociation [54], and loss of emotions [37; 2019 38,55]. One could argue that such negative effects are a matter of practice time and only long-term practices of mindfulness yield enduring positive effects [34,56]. However, Parsons and colleagues [57] found a significant relationship between the amount of long-term practice and negative outcomes such as poorer sleep and cortical arousal. Sahdra and colleagues [58] argue to look at mindfulness not in absolute terms of “*more is better*, *less is worse*.*’*…*rather*, *it is how the different mindfulness skills combine in a person that may be most important for his/her mental health*.” Therefore, the overall impact of mindfulness may depend on personality dispositions and bad effects may be more pronounced for some people but not for others [59].

### 1.3. Mindfulness hurts state-oriented people

Recent findings by Kaufmann and colleagues [17] show that induced mindfulness alienated state-oriented people from the self. More specifically, among state-oriented people, a 90-minute mindfulness training promoted alienation as indicated by a higher tendency to adopt (i.e., falsely self-ascribe) extrinsic goals that were recommended by an expert. Ironically, mindfulness exacerbated the very effect that it was supposed to ameliorate. This supports the idea that “there is no such thing as an unmitigated good” [60].

Our current studies conceptually replicate and extend the research by Kaufmann and colleagues [17] by testing the effects of a 5-minute mindfulness induction, varying stress and no-stress conditions, and applying two different measures of alienation that tap into participants’ preferences and goals [9]. As the self is defined as an extended network of implicit representations of own needs, preferences, and goals, we cover two important aspects of the self: preferences (Study 1) and goals (Study 2).

## 2. Study 1

In Study 1, we tested whether a 5-minute mindfulness exercise leads to alienation from the self among state-oriented participants. As an inverse measure of alienation, we assessed the consistency of preference judgments. If people can reliably access own preferences, they should show some kind of consistency across repeated judgments. However, if people’s access to own preferences is reduced, their judgments should become unreliable and inconsistent [10,61]. According to PSI theory, access to the self is not permanently on or off but changes with different conditions [3,4]. Under adverse compared to neutral conditions, for example, state-oriented participants become more inconsistent when rating art paintings, soft drinks, taste of jam, wallpaper patterns, and Chinese symbols [9,28,62].

In the present study, we used Chinese symbols. We expected state-oriented participants to show lower consistency in repeated judgments of Chinese symbols after the mindfulness exercise compared to the control condition. We did not expect mindfulness to alienate action-oriented participants from their preferences.

### 2.1. Participants

The required number of participants was calculated prior to data collection using G*Power [63]. Kaufmann et al. [17] found small to intermediate interaction effects of Δ*R*^2^ = .07 and .05 in Studies 1 and 2 (*f* = .27 and .23), respectively. To detect a small to intermediate within-between interaction effect of *f* = .20 with a power of .90 and *α* = .05 (ANOVA, 4 groups, 2 measurements), G*Power suggested a minimum of 96 participants. Our final sample consisted of 126 participants (42 men, 84 women) aged 17–86 years (*M* = 31.87, *SD* = 14.84). We recruited participants (42 undergraduates, 48 others) online at a German University.

### 2.2. Methods

**Fig 1:**
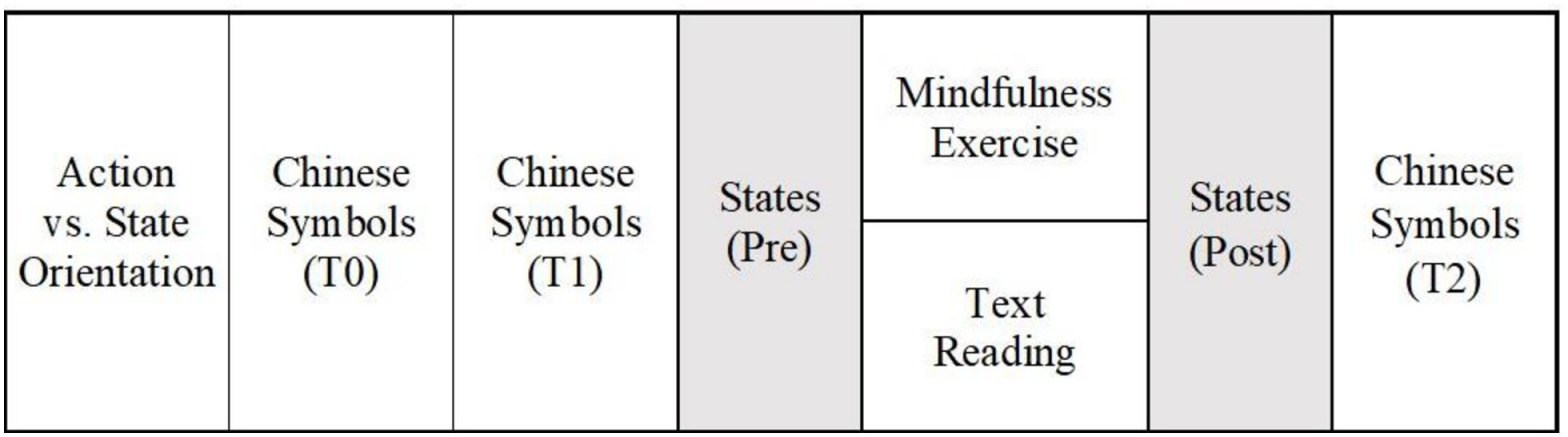
Overview of Study 1.

The ethics committee of the University waived the need for consent. Participants took part in an online experiment. Before starting the experiment, participants gave their consent through a standardized consent form. By clicking the consent field and the ‘continue’ button, participants agreed to participate in the experiment. Additionally, they were informed that they could cancel and revoke their consent and leave the experiment any time as they pleased. As illustrated in Fig 1, participants first completed questionnaires measuring action-state orientation, implicit affect, explicit affect, and state mindfulness (Pre). Next, they rated the attractiveness of 12 Chinese symbols based on their gut feeling on a scale from -9 to +9 (T0). We presented the same 12 Chinese symbols in a new order and asked participants to rate the attractiveness again (T1). Then, we randomly assigned participants to experimental conditions. In the mindfulness group (*n* = 62), participants listened to a 5-minute audio recorded by a local certified mindfulness trainer. The exercise contained steps to draw attention to body sensations of breathing and stay in the present. In the control group (*n* = 64), participants read a paragraph about attention from a textbook by Zimbardo and Gerrig [64] and answered some questions afterwards. Finally, all participants rated their affect and state mindfulness (Post), the Chinese symbols (T2), and some post-experimental questions (e.g., prior experience with mindfulness exercises and Chinese symbols). We thanked and debriefed participants and compensated undergraduate students with course credit. The experiment lasted roughly 30 minutes.

### 2.3. Materials

#### 2.3.1. Action-state orientation

We measured action versus state orientation with the 12 failure-related items of the Action Control Scale (ACS; 8). An example item is: „If I’ve been working on something for four weeks and then everything goes wrong, then (A) it takes a long time to come to terms with it or (B) I don’t think about it much longer”. Option (A) reflects the state-oriented and option (B) the action-oriented response alternative. We counted the number of action-oriented response alternatives (range 0–12). Cronbach’s α = .67 was sufficient for research purposes [65]. Applying the norms [8], participants with values from 0 to 4 were assigned to the state-oriented group (*n* = 56) and participants with values from 5 to 12 to the action-oriented group (*n* = 70). In the absence of multicollinearity (e.g., in experimental designs), a median split is completely legitimate and does not create misleading results [66].

#### 2.3.2. Affect

For the present research question, affect was not relevant (for measures and results, see supplementary material).

#### 2.3.3. State mindfulness

Consistent with Kaufmann and colleagues [17], we adapted five items from the Mindful Attention Awareness Scale (MAAS-K; [34,67]) to measure state rather than trait mindfulness (e.g., “Right now I find it difficult to stay focused on what is happening”, “Right now I find myself doing things without paying attention to them”). Participants rated items on a Likert-scale from 1 (not at all) to 7 (very strong). We reversed items so that higher scores indicate higher state mindfulness (Cronbach’s α = .80/.87 for pre/post measures).

#### 2.3.4. Mindfulness intervention

For the mindfulness intervention, we requested a local certified MBSR trainer to record a 5-minute audio for our experiment. The audio began and ended with a gong. Throughout the exercise, participants were instructed to pay close attention to their breath and to perceive it without changing it and just being in sync with the present moment.

#### 2.3.5. Consistency

We calculated consistency as within-person correlations across repeated preference judgments. For consistency at T1, we correlated participants’ ratings at T0 and T1. For T2, we correlated T0 and T2. Higher consistency indicates reliable access to the self, whereas lower consistency indicates alienation from the self [9,10].

### 2.4. Results

#### 2.4.1. Prior experience

Only 30% of the participants indicated experience with mindfulness exercises (mindfulness condition: 29% vs. control condition: 31%). One participant was a native Chinese speaker, three had some experience with Chinese. The pattern of results was the same when excluding these four participants from the analyses.

#### 2.4.2 Manipulation check

To test the effectiveness of the mindfulness induction, we analyzed state mindfulness using a 2 (Time: pre, post) x 2 (state vs. action orientation) x 2 (mindfulness vs. control condition) ANOVA with repeated measures on the first factor. The significant main effect of Time, *F*(1, 122) = 4.46, *p* < .04, $\eta_{p}^{2}$ = .035, was qualified by a significant Time x Condition interaction, *F*(1, 122) = 6.19, *p* < .02, $\eta_{p}^{2}$ = .048. In the mindfulness condition, there was a significant increase in state mindfulness after the exercise: *M*_pre_ = 4.71 (1.34) vs. *M*_post_ = 5.01 (1.38), *t*(62) = -2.77, *p* < .01. In the control condition, state mindfulness did not change over time: *M*_pre_ = 4.65 (1.40) vs. *M*_post_ = 4.64 (1.41); *t*(64) = 0.09, *p* = .93. There were no differential effects, indicating that the mindfulness induction was equally effective for both state- and action-oriented participants.

#### 2.4.3. Consistency

To test our hypothesis, we analyzed consistency scores using a 2 (Time: pre, post) x 2 (state vs. action orientation) x 2 (mindfulness vs. control condition) ANOVA with repeated measures on the first factor. There was a significant main effect of Time, *F*(1, 122) = 6.99, *p* < .01, $\eta_{p}^{2}$ = .054, indicating that consistency declined over time: *M*_T1_ = .66 (.27) vs. *M*_T2_ = .62 (.28). Consistent with expectations, there was a significant Time x Orientation x Condition interaction, *F*(1, 122) = 5.01, *p =* .027, $\eta_{p}^{2}$ = .039. Note that the three-way interaction remained significant when excluding participants experienced in Chinese, *F*(1, 118) = 5.29, *p =* .023, $\eta_{p}^{2}$ = .043, and when converting correlation coefficients *r* into *z* scores using Fisher transformation to better ensure normality of the dependent variable, *F*(1, 122) = 5.79, *p =* .018, $\eta_{p}^{2}$ = .045. As listed in Table 1, in the mindfulness condition, state-oriented participants showed a significant decrease in consistency, *t*(23) = 3.10, *p* < .01, whereas action-oriented participants did not, *t*(37) = 0.18, *p* = .86. In the control condition, in contrast, state-oriented participants did not change in consistency, *t*(31) = 0.19, *p* = .85, whereas action-oriented participants showed a significant decrease, *t*(31) = 2.83, *p* < .01.

**Table 1:** Means (and Standard Deviations) of Consistency (Within-Person Correlations) in Preference Judgments in Study 1 (N = 126).

	Consistency		
	*r*_Pre_	*r*_Post_	Δ*r*
Control Condition			
State Orientation	.62 (.29)	.61 (.31)	-.01 (.28)
Action Orientation	.73^a^ (.24)	.66^b^ (.27)	-.07 (.14)
Mindfulness Condition			
State Orientation	.75^a^ (.12)	.63^b^ (.18)	**-.13** (.20)
Action Orientation	.60 (.31)	.59 (.32)	**-.01** (.24)

*Note*. Different superscripts indicate significant differences in paired *t*-Tests (*p* < .05). Means printed in bold significantly differ in independent *t*-Tests (*p* < .05).

**Fig 2:**
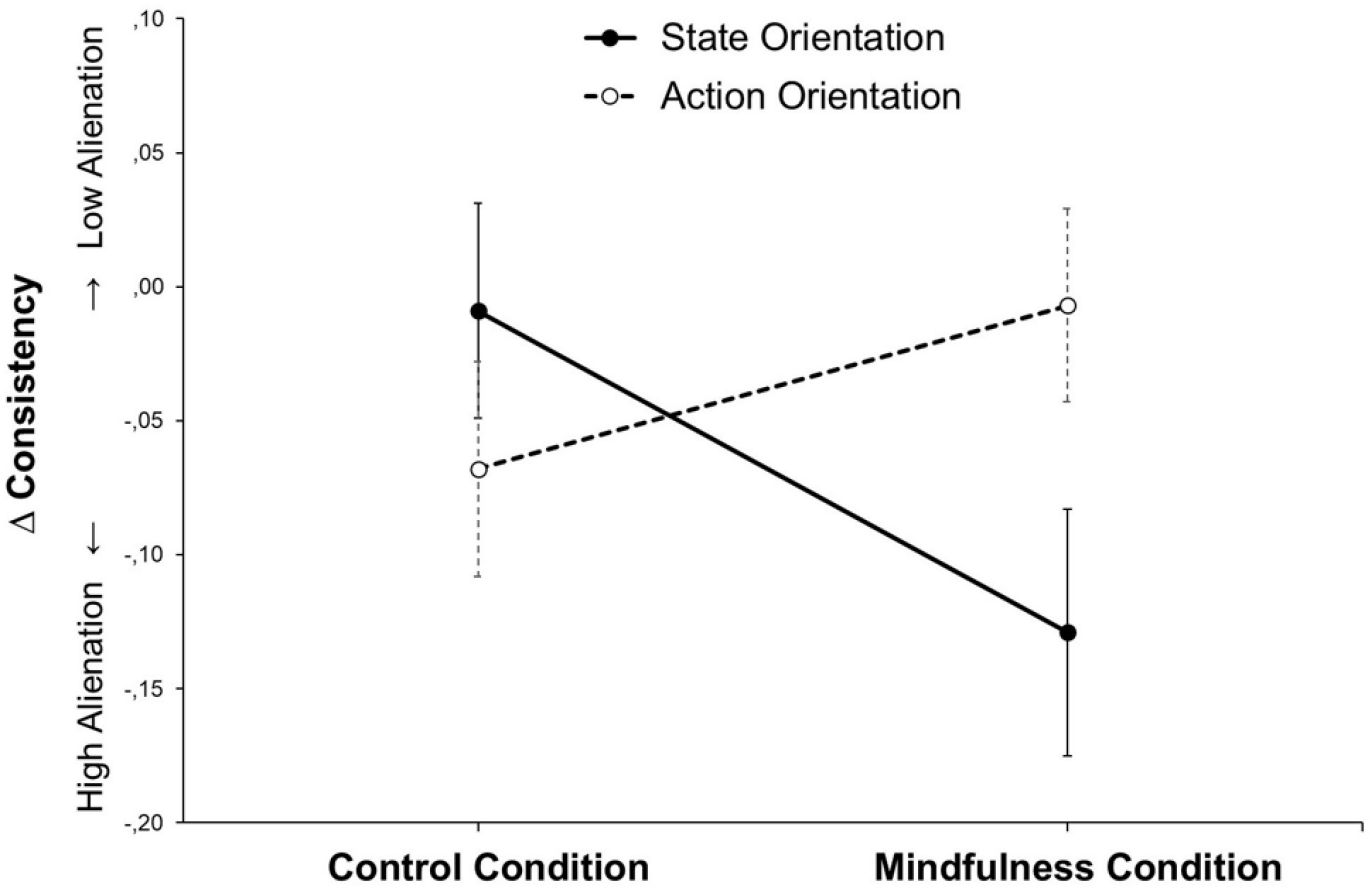
Pre-post changes in the consistency of preference judgments (within-person correlations: *r*_Post_−*r*_Pre_) in Study 1 (*N* = 126).

To illustrate the effect (see Fig 2), we calculated change scores (*r*_post_—*r*_pre_). State-oriented participants in the mindfulness condition showed a marginally stronger decrease in consistency compared to state-oriented participants in the control condition, *t*(54) = 1.76, *p* = .084, and a significantly stronger decrease compared to action-oriented participants in the mindfulness condition, *t*(60) = -2.08, *p* = .042.

### 2.5. Discussion

In line with our hypothesis, state-oriented participants showed lower consistency across preference judgments in the mindfulness compared to the neutral condition and in comparison, with their action-oriented counterparts. The finding indicates that state-oriented people became alienated from their preferences after the mindfulness induction. One could question whether the alienating effect for state-oriented people extends beyond preferences. In Study 2, we therefore broadened our scope from preferences to goals. In addition, we included a stress induction because people often seek mindfulness as a stress reduction tool [68,69].

## 3. Study 2

In Study 2, we aimed to replicate and extend the findings of Study 1. First, we induced stress in all participants. Second, we used a different measure of alienation that taps into the adoption of goal recommendations. People have to deal with social expectations on a daily basis and may adopt recommendations wholeheartedly, halfheartedly, or not at all—indicating the degree of integration into the self [70]. Intrinsic goals can be easily integrated into the self because they fulfill inner needs. Extrinsic goals, in contrast, are diametrically opposed to inner needs and often introjected rather than integrated [71]. However, extrinsic goals are not bad per se but a lower tendency to adopt intrinsic over extrinsic goals is detrimental to psychological health [72].

In the present study, we used false self-ascriptions of recommended goals to measure goal adoption [17] and looked at the relation between intrinsic and extrinsic goals rather than just extrinsic goals [72]. We expected state-oriented participants to show a lower tendency to adopt (falsely self-ascribe) intrinsic over extrinsic goal recommendations after the mindfulness exercise compared to the control condition. We did not expect mindfulness to alienate action-oriented participants from their goals.

### 3.1. Participants

The required number of participants was calculated prior to data collection using G*Power [63]. Consistent with Kaufmann et al. [17], we observed a small to intermediate interaction effect in Study 1 ($\eta_{p}^{2}$ = .039; *f* = .20). To detect a small to intermediate within-between interaction effect of *f* = .20 with a power of .90 and *α* = .05 (ANOVA, 4 groups, 2 measurements), the result suggested a minimum of 96 participants. The sample consisted of 108 people (30 men, 75 women, 3 diverse) aged 17–69 years (*M* = 28.00, *SD* = 9.84). We recruited participants online at a German University (21 undergraduates, 87 others).

### 3.2. Methods

**Fig 3:**
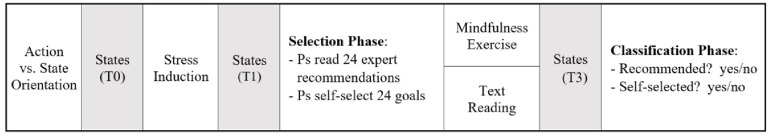
Overview of Study 2.

The ethics committee of the University waived the need for consent. Participants took part in an online assessment. As in Study 1, before starting the experiment, participants gave their consent to participate in the experiment by clicking the consent field and the ‘continue’ button. Additionally, they were informed that they could cancel and revoke their consent and leave the experiment at any time. As illustrated in Fig 3, participants first completed measures of action-state orientation, implicit/explicit affect, and state mindfulness (T1).

#### 3.2.1. Stress induction

Next, we induced stress in all participants through an imagery exercise which has been successfully used in previous work (24, Exp. 3; 51): In a 4-minute audio, participants were instructed to revitalize an experience with a threatening person, the person’s tone of voice and words, and their own feelings in that situation. Afterwards, we measured implicit/explicit affect and state mindfulness again (T2).

#### 3.2.2. Selection phase

We presented 24 intrinsic and 24 extrinsic goals (see S2 and S3 Tables in S1 File). *Expert recommendations*: Twelve intrinsic and twelve extrinsic goals were marked with an asterisk to indicate that experts recommended these goals because they allegedly matched participants’ initial personality screening. We asked participants to read each goal aloud and confirm the recommendation status by pressing a corresponding key. *Self-selection*: We presented eight lists each with six goals that were homogeneous with respect to goal type and recommendation status. We asked participants to select three goals of each list they would like to pursue. Thus, there were six intrinsic and six extrinsic goals in each of the following categories: both (recommended and self-selected), recommended, self-selected, remaining (see S1 Table in S1 File).

#### 3.2.3. Experimental induction

We randomly assigned participants to the mindfulness group (*n* = 56) versus control group (*n* = 52). The mindfulness exercise and text-reading task were the same as in Study 1. Next, we measured implicit/explicit affect and state mindfulness (T3).

#### 3.2.4. Classification phase

In two separate tasks, participants needed to classify goals as recommended (yes vs. no) and as self-selected (yes vs. no). Finally, participants answered some post-experimental questions. We thanked and debriefed participants and compensated undergraduate students with course credit. The experiment lasted roughly 45 minutes.

### 3.3. Materials

We used the same measures as in Study 1 for action orientation (Cronbach’s α = .74), affective states (explicit negative affect: .75/.86/.74), and state mindfulness (.82/.83/.86). For the dependent variable, we calculated the percentage of *recommended goals* that participants falsely ascribed as self-selected, separately for intrinsic and extrinsic goals. For example, an intrinsic goal adoption rate of 33% would indicate that participants falsely ascribed two out of six recommended intrinsic goals as self-selected. Finally, we calculated differences scores: intrinsic minus extrinsic goal adoption rates. High values indicate a healthy tendency to adopt recommended goals more often when they were intrinsic rather than extrinsic (i.e., low alienation), whereas low values indicated that people did not consider the goal type when adopting recommended goals (i.e., high alienation).

### 3.4. Results

#### 3.4.1. Prior experience

Only 29% of the participants indicated experience with mindfulness exercises (mindfulness condition: 23% vs. control condition: 35%; *t*(106) = 1.31, *p* = .194).

#### 3.4.2. Manipulation check

To test the effectiveness of our inductions, we conducted two separate 3 (time: T1, T2, T3) x 2 (state vs. action orientation) x 2 (mindfulness vs. control condition) ANOVAs with repeated measures on the first factor.

The analysis of *explicit negative affect* yielded only a significant main effect of Time, *F*(2, 208) = 39.97, *p* < .001, $\eta_{p}^{2}$ = .278, indicating that explicit negative affect significantly increased after the stress induction (*M*_*T1*_ = 1.49 (0.58) vs. *M*_*T2*_ = 1.99 (0.82); *p* < .001) and returned to baseline afterwards (*M*_*T3*_ = 1.40 (0.54)). There were no differential effects indicating that the stress induction was equally effective for both state- and action-oriented participants (see also S4 and S5 Tables in S1 File).

The analysis of *state mindfulness* yielded a significant main effect of Time, *F*(2, 208) = 4.21, *p* < .02, $\eta_{p}^{2}$ = .039, that was qualified by a significant Time x Condition interaction, *F*(2, 208) = 7.44, *p* < .001, $\eta_{p}^{2}$ = .067. As listed in Table 2, in the mindfulness condition, state mindfulness was significantly higher at T3 compared to T1 and T2. In the control condition, in contrast, state mindfulness was not significantly higher at T3 compared to T1 and T2. There were no main or interaction effects of state orientation indicating that the mindfulness induction was equally effective for both state- and action-oriented participants.

**Table 2:** Means (and Standard Deviations) of State Mindfulness in Study 2 (N = 108).

	Stress ↓		Exercise ↓	
	T1	T2		T3
State Mindfulness				
Control Condition	4.99^a^ (1.34)	4.67^b^ (1.34)		4.74 (1.38)
Mindfulness Condition	4.63^a^ (1.35)	4.66^a^ (1.37)		5.23^b^ (1.24)

*Note*. Different superscripts indicate significant within-subject contrasts.

#### 3.4.3. False self-ascriptions

To test our hypothesis, we used false self-ascription rates of recommended goals as a dependent variable and conducted a 2 (intrinsic vs. extrinsic goals) x 2 (state vs. action orientation) x 2 (mindfulness vs. control condition) ANOVA with repeated measures on the first factor. The highly significant main effect of Goal Type, *F*(1, 104) = *F*(1, 104) = 19.69, *p* < .001, $\eta_{p}^{2}$ = .159, indicated that participants adopted (falsely self-ascribed) more recommendations of intrinsic compared to extrinsic goals: *M*_*intrinsic*_ = 46.45% (24.65) vs. *M*_*extrinsic*_ = 32.87% (26.13). More importantly, the Goal Type x Orientation x Condition interaction was significant, *F*(1, 104) = 4.89, *p* = .029, $\eta_{p}^{2}$ = .045 (see Table 3).

**Table 3:** Means (and Standard Deviations) of the Tendency to Adopt (Falsely Self-Ascribe) Recommended Goals (in %) in Study 2 (N = 108).

	Recommended Goals		
	Intrinsic Goals	Extrinsic Goals	Diff_(Intr-Extr)_
Control Condition			
State Orientation	46.30^a^ (29.36)	30.25^b^ (26.97)	16.05 (33.49)
Action Orientation	42.00 (21.58)	32.67 (24.76)	9.33 (32.30)
Mindfulness Condition			
State Orientation	43.59 (22.15)	39.74 (24.53)	**3.85** (24.18)
Action Orientation	52.78^a^ (24.40)	29.44^b^ (27.92)	**23.33** (31.74)

*Note*. Different superscripts indicated significant differences in paired *t*-Tests (*p* < .05). Means printed in bold significantly differ in independent *t*-Tests (*p* < .05).

To facilitate interpretation of the three-way interaction, we analyzed both experimental conditions separately. In the mindfulness condition, the significant main effect of Goal Type, *F*(1, 54) = 12.68, *p* < .001, $\eta_{p}^{2}$ = .190, was qualified by a significant Goal Type x Orientation interaction, *F*(1, 54) = 6.52, *p* < .015, $\eta_{p}^{2}$ = .108. In contrast to action-oriented participants, state-oriented participants did not adopt more recommendations of intrinsic compared to extrinsic goals. In the control condition, the Goal Type x Orientation ANOVA yielded only a significant main effect of Goal Type, *F*(1, 50) = 7.72, *p* < .008, $\eta_{p}^{2}$ = .134, but no significant Goal Type x Orientation interaction, *F*(1, 50) = 0.54, *p* = .466, $\eta_{p}^{2}$ = .011, indicating that both state- and action-oriented participants adopted more recommendations of intrinsic compared to extrinsic goals.

**Fig 4:**
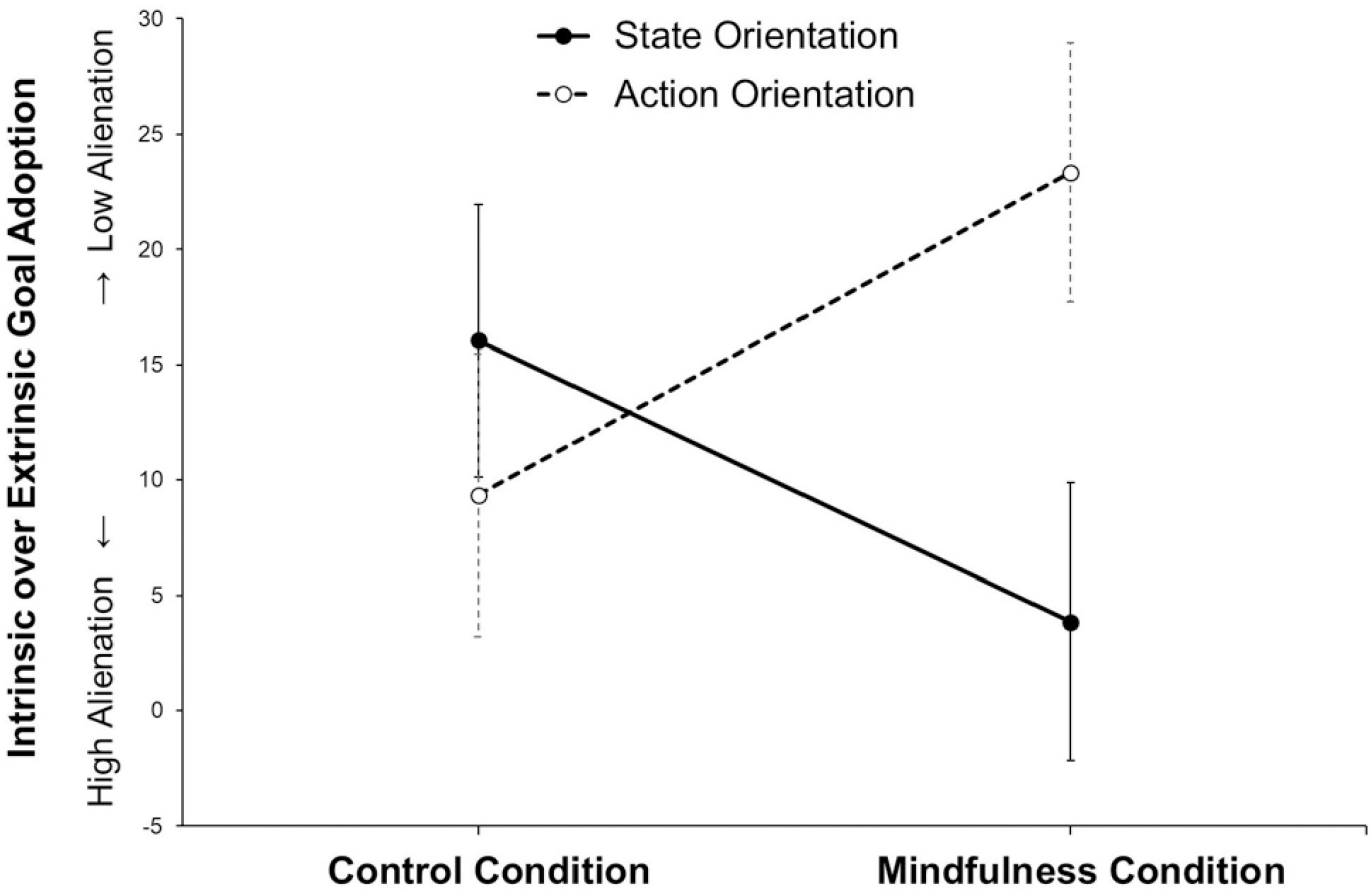
Alienation as assessed by a lower tendency to adopt (falsely self-ascribe) intrinsic over extrinsic goal recommendations in Study 2 (*N* = 108).

To illustrate the finding, we used difference scores of intrinsic minus extrinsic goal adoption (see Fig 4). In the mindfulness condition, state- compared to action-oriented participants showed a significantly lower tendency to adopt intrinsic over extrinsic goals: *M*_State_ = 3.85% vs. *M*_Action_ = 23.33%, *t*(54) = -2.55, *p* = .014. In the control condition, state- and action-oriented participants did not differ in the tendency to adopt intrinsic over extrinsic goals: *M*_State_ = 16.05% vs. *A*_ction_ = 9.33%, *t*(50) = 0.74, *p* = .466.

### 3.5. Discussion

In Study 2, state-oriented participants showed a lower tendency to adopt intrinsic over extrinsic goal recommendations in mindfulness compared to the control condition. This means that state-oriented people showed alienation from self-congruent goals after the mindfulness induction. Thus, our results replicated with a different measure of alienation and with the addition of a stress induction. This further supports our assumption that practicing mindfulness alienated state-oriented people from the self with and without stress. In both studies, we did not find alienating effects among action-oriented participants.

## 4. General discussion

Mindfulness is a means to self-reflection and awakening and many findings support this claim [73,74]. Contrarily, in the current research, we empirically demonstrated that practicing mindfulness has adverse effects and alienates some people from their self. In two studies, we showed that, among state-oriented participants, mindfulness promoted alienation as measured by lower consistency of preference judgments and a lower tendency to adopt intrinsic over extrinsic goals. This alienating effect of mindfulness was absent in action-oriented participants.

Mindfulness not only detached state-oriented people from their preferences but also from their intrinsic goals. This could render into poor psychological functioning for state-oriented people affecting their everyday life choices and decisions and augmenting their self-regulatory deficit [11]. Although higher false self-ascriptions of assigned goals and lower consistency in repeated preference judgments do not seem like a big problem in themselves, they indicate a tendency towards alienation that has a real-life impact. For example, such markers of alienation from the *self* have been associated with higher depression and anxiety [75], higher cortisol reactivity [76], higher medication intake [77], lower flow experience [78], and lower work performance [79]. Therefore, our finding that mindfulness increases the tendency towards alienation among state-oriented people has profound consequences for their psychological functioning across many life domains. Note that our pattern of findings was consistent across two different samples, two different and well-established measures of alienation, and two different conditions of induced stress versus no stress. This methodological convergence increases confidence in our findings.

Our findings extend previous research on mindfulness and the self in at least three important ways. First, we took a differential view and tested for whom the practice is effective, ineffective, or even counter-indicated [58,80,81]. This is important because state-oriented people are the ones who may actually need and seek mindfulness the most in order to gain better self-access [29]. Previous findings suggest that depressed people and ruminators, for example, do indeed profit from mindfulness interventions [82–84]. In contrast, our studies showed the opposite effect. State-oriented people did not only not profit but incurred harm. These detrimental effects on self-related variables may have gone unnoticed in previous studies because research relies heavily on self-report [85–87].

Second, we measured self-access beyond self-report. Although self-report measures contribute well to the overall understanding of psychological constructs, for a mechanism like access to the implicit self, results are more credible when applying non-reactive measures [88,89]. Positive effects of mindfulness often emerge when measuring self-related variables by self-report [90]. However, the present findings show that the effects reverse when measuring self-access non-reactively (see also 17). Hence, self-reports should be supplemented with more ‘objective’ behavioral methods.

Third, Kaufmann and colleagues [17] tested the alienating effect for state-oriented people only under stress. The present findings show that mindfulness alienated state-oriented people across stress and no stress conditions. This has important consequences for understanding *why* mindfulness has alienating effects. On the basis of PSI theory [3,4,11], Kaufmann and colleagues [17] argued that practicing mindfulness might activate a basic processing system –called *object recognition*–which is associated with attentional focus to isolated objects and sensations (e.g., breathing, heartbeat; [91]) and antagonistically related to the self. This means that any activation of object recognition will reduce access to the self if it is not actively regulated. The current findings suggest that mindfulness might also activate another basic processing system–called *intuitive behavior control*–that is responsible for behavioral routines and staying in the ‘here and now’. Because it is antagonistically related to the self, any activation of intuitive behavior control will also reduce access to the self. Intuitive behavior control can be activated through non-conscious, indirect cues (e.g., priming; [92]), which is similar to the instructions given in mindfulness training for being in the present. Whereas object recognition is associated with negative affect, intuitive behavior control is associated with positive affect [3]. Activating this basic processing system might lead state-oriented people to *‘mindfully and happily miss themselves’* without conscious realization. However, the possible routes into alienation need further investigation.

## 5. Limitations and future directions

The present research is not free from shortcomings. First, both studies had short mindfulness interventions as even short exercises have positive impacts on psychological functioning [93–95]. However, the outcomes could differ with longer mindfulness inductions. Long-term practice of mindfulness, for example, might help state-oriented people to develop the very self-regulatory abilities whose lack resulted in alienation.

Second, our participants are mostly amateur practitioners of mindfulness and might not have the “right” motivation to practice mindfulness. The short-term challenges of mindfulness for naïve practitioners are well-known (e.g., [37]) as well as the effects of motivation to practice on the practice outcomes [96]. Trying mindfulness without desire to practice might not have the same effects as a genuine mindfulness practice. Therefore, the outcomes might differ for advanced practitioners [97,98].

Finally, our findings draw attention towards a need to adapt mindfulness trainings in accordance with individual differences to enable state-oriented people to profit from its well-documented benefits. For example, state-oriented people profit from social support—even if it is imagined [12,24,99]. In future research, participants could imagine practicing the mindfulness exercise together with a close friend to integrate the positive effects of social support.

## 6. Conclusion

Mindfulness meditation is in vogue as a self-help tool for its profound psychological benefits across different populations and conditions [100,101]. One of the great potentials of mindfulness is that it helps people to get *in touch* with themselves [86]. However, whether mindfulness is beneficial or not depends on people’s personality dispositions. In the present studies, we show that mindfulness had the exact opposite effect and caused some people to get *out of touch* from their self. Although participants reported being mindful, on a deeper level, the practice alienated state-oriented people from their self. In conclusion, state-oriented people may mindfully miss themselves.

## Supporting information

S1 File All the supporting information can be found in S1 File. (DOCX)
