# Supplementary material for: Mindfully missing myself: Induced mindfulness causes alienation among poor self-regulators
Source: PLoS One. 2024 May 21;19(5):e0303505. doi: 10.1371/journal.pone.0303505 (PMC11108221; doi:10.1371/journal.pone.0303505)
Supplement: S1 File — (DOCX) [file pone.0303505.s001.docx]

**1. Study 1**

**1.1. Materials**

**1.1.1. Implicit affect**

We administered the Implicit Positive Affect and Negative Affect Test (IPANAT; Quirin et al., 2009) before (pre) and after (post) the mindfulness versus control induction. Participants rated the extent to which artificial words (e.g. SAFME, VIKES) from a putative artificial language express positive (happy, cheerful, energetic) and negative emotion words (helpless, tense, inhibited) on a scale from 1 (doesn’t fit at all) to 4 (fits very well). For each single affect item, we averaged ratings across the six artificial words. Next, we computed an implicit affect score by averaging across the three positive (Cronbach’s α = .72/.80) and the three negative affect items (Cronbach’s α = .66/.77) at pre and post, respectively.

**1.1.2. Explicit affect**

Three positive (happy, cheerful, joyful; Cronbach’s α = .75/.74) and three negative items (helpless, clueless, inhibited; Cronbach’s α = .79/.83) were used at T1 and T2. Participants rated the extent to which the statement applied to them ‘at the present moment’ using a scale from 1 (not at all) to 4 (very much).

**1.2. Results**

**1.2.1. Affective states**

We conducted four separate 2 (Time: pre, post) x 2 (Orientation: state vs. action orientation) x 2 (Condition: mindfulness vs. control condition) ANOVAs with repeated measures on the first factor (see Table S1).

*Implicit negative affect.* The analysis yielded a significant main effect of Time, *F*(1, 122) = 9.71, *p =* .002, $\eta_{p}^{2}$ = .074, that was qualified by a significant Time x Condition interaction, *F*(1, 122) = 11.18, *p =* .001, $\eta_{p}^{2}$ = .084. Implicit negative affect remained stable in the control condition but significantly decreased in the mindfulness condition.

*Explicit negative affect.* The analysis yielded only a significant Time x Condition interaction, *F*(1, 122) = 10.51, *p =* .002, $\eta_{p}^{2}$ = .079. Explicit negative affect remained stable in the control condition but significantly decreased in the mindfulness condition.

*Implicit positive affect.* The analysis yielded only a significant Time x Condition interaction, *F*(1, 122) = 4.12, *p =* .044, $\eta_{p}^{2}$ = .033. Implicit positive affect significantly decreased in the control condition but remained stable in the mindfulness condition.

*Explicit positive affect.* The analysis yielded no significant effects. Dependent t-Tests indicated that explicit positive affect decreased in the control condition, but remained stable in the mindfulness condition. However, the Time x Condition interaction was not significant, *F*(1, 122) = 1.64, *p =* .203, $\eta_{p}^{2}$ = .013.

Dispositional action versus state orientation did not show significant main or interaction effects in any of the analyses, indicating that the mindfulness induction was equally effective for both state- and action-oriented participants.

Table S1. *Means (and Standard Deviations) of Affective States in Study 1 (N = 126)*

|  | Exercise ↓ | |  |
| --- | --- | --- | --- |
|  | Pre | Post | |
| Implicit Negative Affect |  |  | |
| Control Condition | 1.78 (0.38) | 1.79 (0.44) | |
| Mindfulness Condition | 1.77^a^ (0.46) | 1.56^b^ (0.44) | |
| Explicit Negative Affect |  |  | |
| Control Condition | 1.41 (0.54) | 1.48 (0.64) | |
| Mindfulness Condition | 1.46^a^ (0.59) | 1.33^b^ (0.51) | |
| Implicit Positive Affect |  |  | |
| Control Condition | 2.30^a^ (0.52) | 2.18^b^ (0.54) | |
| Mindfulness Condition | 2.25 (0.43) | 2.27 (0.48) | |
| Explicit Positive Affect |  |  | |
| Control Condition | 2.41^a^ (0.66) | 2.30^b^ (0.73) | |
| Mindfulness Condition | 2.41 (0.64) | 2.40 (0.64) | |

*Note.* Different superscripts indicate significant within-subject contrasts.

**2. Study 2**

**2.1. Materials**

**2.1.1. Affect**

In the present study, internal consistencies at T1/T2/T3 were sufficient for implicit positive affect (Cronbach’s α = .70/.85/.80), implicit negative affect (Cronbach’s α = .64/.88/.83), and explicit positive affect (Cronbach’s α = .82/.89/.84).

**2.1.2. Goal selection and classification**

We used the Process-Analytic Neuroticism Test for Adults (PANTER; Adults = ′*Erwachsene*′ in German) by Kuhl and Kazén (1994; see also Baumann & Kuhl, 2003; Kazén, Baumann & Kuhl, 2003). Table S2 illustrates the goal selection and goal classification phases in the PANTER. Table S3 presents the list of 48 goals.

Table S2. *Goal Selection and Classification Phases in Study 2*

|  |  |  |  | **Reported Source** | |
| --- | --- | --- | --- | --- | --- |
|  | Recom-mended | Self-Selected | **Objective Source** | **Recommended** | **Self-Selected** |
| 24 Intrinsic | 12 yes | 6 yes | Both | Correct | Correct |
|  |  | 6 no | Recommended | Correct | False Self-Ascription |
|  | 12 no | 6 yes | Self-selected | False Other-Ascription | Correct |
|  |  | 6 no | Remaining | False Other-Ascription | False Self-Ascription |
| 24 Extrinsic | 12 yes | 6 yes | Both | Correct | Correct |
|  |  | 6 no | Recommended | Correct | False Self-Ascription |
|  | 12 no | 6 yes | Self-selected | False Other-Ascription | Correct |
|  |  | 6 no | Remaining | False Other-Ascription | False Self-Ascription |

*Note.* False self-ascription rates of recommended goals: intrinsic > extrinsic = self-access; intrinsic ≤ extrinsic = alienation.

Table S3: *List of 48 Goals in Study 2 (* Recommended by Experts)*

| **Intrinsic Goals** | **Extrinsic Goals** |
| --- | --- |
| **Community Involvement** | **Fame** |
| 1. * helping people in need without expecting anything in return  *(bedürftigen Menschen helfen, ohne etwas dafür zu erwarten)* | 1. * be admired by many people  *(von vielen Personen bewundert werden)* |
| 1. * help others improve their lives *(anderen helfen, ihr Leben zu verbessern)* | 1. become better known and more popular  *(bekannter und beliebter werden)* |
| 1. trying to make the world a better place *(versuchen, aus der Welt einen besseren Ort zu machen)* | 1. * be respected by other people  *(bei anderen Personen angesehen sein)* |
| 1. do volunteer work *(ehrenamtliche Arbeit leisten)* | 1. have a large circle of friends  *(einen großen Bekanntenkreis haben)* |
| 1. * get involved in a community *(mich für eine Gemeinschaft engagieren)* | 1. * have high social status  *(einen hohen sozialen Status haben)* |
| 1. * encourage and support other people *(andere Menschen fördern und unterstützen)* | 1. * have a respected place in society  *(angesehenen Platz in der Gesellschaft haben)* |
| 1. give advice to other people *(anderen Menschen Rat geben)* | 1. attain an influential position in society  *(eine gesellschaftlich einflussreiche Position erreichen)* |
| 1. take responsibility for others *(Verantwortung für andere übernehmen)* | 1. associate with celebrities  *(mit Berühmtheiten verkehren)* |
| **Personal Growth** | **Financial Success** |
| 1. decide for myself what I do instead of going with the flow *(selbst entscheiden, was ich tue, anstatt mit dem Strom zu schwimmen)* | 1. * finding ways to buy anything I want  *(Wege finden, alles zu kaufen, was ich möchte)* |
| 1. * know and accept who I really am *(wissen und akzeptieren, wer ich wirklich bin)* | 1. start a high-paying career  *(eine hochbezahlte Karriere starten)* |
| 1. gain more insight into myself and my behavior *(mehr Einsicht in mich selbst und mein Verhalten erlangen)* | 1. * work to get a well-paying job  *(daran arbeiten, einen gutbezahlten Job zu bekommen)* |
| 1. * constantly evolving and learning new things *(mich ständig weiterentwickeln und neue Dinge lernen)* | 1. * increase my property  *(meinen Besitz vergrößern)* |
| 1. * find jobs that I can get lost in  *(Tätigkeiten finden, in denen ich aufgehen kann)* | 1. be state of the art in technology *(auf dem neuesten Stand der Technik sein)* |
| 1. gaining experience with many different people *(Erfahrungen mit vielen unterschiedlichen Menschen sammeln)* | 1. own many expensive things  *(viele teure Dinge besitzen)* |
| 1. * deal with things that really interest me *(mich mit Dingen beschäftigen, die mich wirklich interessieren)* | 1. * live in prosperity  *(in Wohlstand leben)* |
| 1. constantly checking and renewing my knowledge *(mein Wissen ständig überprüfen und erneuern)* | 1. belong to the top ten thousand  *(zu den oberen Zehntausend gehören)* |
| **Emotional Intimacy** | **Image** |
| 1. share my life with someone i really love *(mein Leben mit jemandem teilen, den ich wirklich liebe)* | 1. to be stylishly dressed  *(modisch stilsicher gekleidet sein)* |
| 1. * spending time with people who are important to me *(Zeit mit Menschen verbringen, die mir wichtig sind)* | 1. achieve the perfect "look"  *(den perfekten "Look" erreichen)* |
| 1. have intimate relationships with other people *(vertraute Beziehungen zu anderen Menschen haben)* | 1. * getting compliments on my good looks  *(Komplimente über mein gutes Aussehen bekommen)* |
| 1. * have good friends that I can really count on *(gute Freunde haben, auf die ich wirklich zählen kann)* | 1. build an image that others admire  *(ein Image aufbauen, das andere bewundern)* |
| 1. * communicate personally with other people *(mich mit anderen Menschen persönlich austauschen)* | 1. make the most of my looks  *(das Beste aus meinem Aussehen machen)* |
| 1. feel close to other people *(mich anderen Menschen nah fühlen)* | 1. * have a great charisma  *(eine tolle Ausstrahlung besitzen)* |
| 1. lead a deep, trusting partnership *(eine tiefe, vertrauensvolle Partnerschaft führen)* | 1. * be perceived as particularly attractive  *(als besonders anziehend empfunden werden)* |
| 1. * to let your guards down with someone *(mich bei jemandem fallen lassen können)* | 1. * be admired by the opposite sex  *(vom anderen Geschlecht bewundert werden)* |

**2.2. Results**

**2.2.1. Affective states**.

We conducted four separate 3 (Time: T1, T2, T3) x 2 (Orientation: state vs. action orientation) x 2 (Condition: mindfulness vs. control condition) ANOVAs with repeated measures on the first factor (see Table S4).

*Implicit negative affect.* The analysis yielded only a significant main effect of Time, *F*(2, 208) = 37.51, *p* < .001, $\eta_{p}^{2}$ = .265. In both conditions, implicit negative affect increased after the stress induction and decreased below baseline level after the mindfulness and text reading exercises.

*Explicit negative affect.* The analysis yielded only a significant main effect of Time, *F*(2, 208) = 39.97, *p* < .001, $\eta_{p}^{2}$ = .278. In both conditions, explicit negative affect increased after the stress induction and decreased afterwards. Within-subject contrasts indicate that explicit negative affect returned to baseline in the control condition, whereas it decreased below baseline in the mindfulness condition. However, the Time x Condition interaction was not significant, *F*(2, 208) = .76, *p =* .467, $\eta_{p}^{2}$ = .007.

*Implicit positive affect.* The analysis yielded only a significant main effect of Time, *F*(2, 208) = 23.97, *p <* .001, $\eta_{p}^{2}$ = .187. In both conditions, implicit positive affect significantly decreased after the stress induction and returned to baseline level afterwards.

*Explicit positive affect.* The analysis yielded a significant main effect of Time, *F*(2, 208) = 31.98, *p <* .001, $\eta_{p}^{2}$ = .235, that was qualified by a significant Time x Orientation x Condition interaction, *F*(2, 208) = 4.32, *p =* .015, $\eta_{p}^{2}$ = .040. The effect is listed in Table S5. To better understand the three-way interaction, we calculated two separate Time x Orientation ANOVAs for each condition, respectively. In the control condition, the Time x Orientation interaction was not significant, *F*(2, 100) = 0.82, *p =* .442, $\eta_{p}^{2}$ = .016, indicating that both state- and action-oriented participants restored explicit positive affect back to baseline level after the text-reading exercise. In the mindfulness condition, in contrast, the Time x Orientation interaction was significant, *F*(2, 108) = 5.11, *p =* .028, $\eta_{p}^{2}$ = .086, indicating that only state-oriented but not action-oriented participants restored explicit positive affect back to baseline level after the mindfulness exercise.

Table S4. *Means (and Standard Deviations) of Affective States in Study 2 (N = 108).*

|  | Stress ↓ | | Exercise ↓ | |
| --- | --- | --- | --- | --- |
|  | T1 | T2 | | T3 |
| Implicit Negative Affect |  |  | |  |
| Control Condition | 1.75^a^ (0.35) | 1.97^b^ (0.53) | | 1.65^c^ (0.44) |
| Mindfulness Condition | 1.87^a^ (0.37) | 2.15^b^ (0.62) | | 1.71^c^ (0.47) |
| Explicit Negative Affect |  |  | |  |
| Control Condition | 1.50^a^ (0.62) | 2.04^b^ (0.82) | | 1.50^a^ (0.59) |
| Mindfulness Condition | 1.48^a^ (0.55) | 1.95^b^ (0.83) | | 1.31^c^ (0.47) |
| Implicit Positive Affect |  |  | |  |
| Control Condition | 2.23^a^ (0.47) | 1.95^b^ (0.47) | | 2.22^a^ (0.53) |
| Mindfulness Condition | 2.30^a^ (0.42) | 2.07^b^ (0.58) | | 2.28^a^ (0.41) |
| Explicit Positive Affect |  |  | |  |
| Control Condition | 2.33^a^ (0.76) | 1.92^b^ (0.76) | | 2.21^a^ (0.82) |
| Mindfulness Condition | 2.43^a^ (0.63) | 2.15^b^ (0.68) | | 2.35^a^ (0.67) |

*Note.* Different superscripts indicate significant within-subject contrasts.

Taken together, the mindfulness induction did not work less effectively for state- compared to action-oriented participants. To the contrary, according to self-report, the mindfulness exercise was even more effective for state- compared to action-oriented participants in restoring positive affect.

Table S5. *Means (and Standard Deviations) of Explicit Positive Affect in Study 2 (N = 108).*

|  | Stress ↓ | | Exercise ↓ | |
| --- | --- | --- | --- | --- |
|  | T1 | T2 | | T3 |
| Control Condition |  |  | |  |
| State Orientation | 2.46^a^ (0.80) | 1.98^b^ (0.67) | | 2.26^a^ (0.81) |
| Action Orientation | 2.20^a^ (0.71) | 1.87^b^ (0.87) | | 2.15^a^ (0.84) |
| Mindfulness Condition |  |  | |  |
| State Orientation | 2.36^a^ (0.66) | 2.15^b^ (0.71) | | 2.47^a^ (0.65) |
| Action Orientation | 2.49^a^ (0.60) | 2.14^b^ (0.66) | | 2.23^b^ (0.67) |

*Note.* Different superscripts indicate significant within-subject contrasts.

Table S6. *Means (in %) and Standard Deviations (in Parentheses) of the Tendency to Falsely Self-Ascribe Remaining Goals in Study 2 (N = 108).*

|  | Remaining Goals | | |
| --- | --- | --- | --- |
|  | Intrinsic Goals | Extrinsic Goals | Diff_(Intr-Extr)_ |
| Control Condition |  |  |  |
| State Orientation | 31.48^a^(28.24) | 17.28^b^(27.92) | 14.20 (29.13) |
| Action Orientation | 29.33^a^(21.13) | 10.00^b^(17.35) | 19.33 (25.77) |
| Mindfulness Condition |  |  |  |
| State Orientation | 32.69^a^(24.26) | 15.38^b^ (16.95) | 17.31 (26.45) |
| Action Orientation | 35.00^a^(23.31) | 17.22^b^(23.77) | 17.78 (24.73) |

*Note.* Different superscripts indicate significant differences dependent *t*-Tests (*p* < .05). Means printed in bold are significantly different in independent *t*-Tests (*p* < .05).

**Supplementary References**

Quirin, M., Kazén, M., & Kuhl, J. (2009). When nonsense sounds happy or helpless: the implicit positive and negative affect test (IPANAT). *Journal of Personality and Social Psychology,* 97(3), 500-516. [https://doi.org/10.1037/a0016063](https://psycnet.apa.org/doi/10.1037/a0016063)
